# Underwater endoscopic submucosal dissection using gel immersion for early gastric cancer with situs inversus totalis

**DOI:** 10.1055/a-2541-2073

**Published:** 2025-03-03

**Authors:** Takahiro Muramatsu, Masakatsu Fukuzawa, Takashi Kawai, Midori Mizumachi, Yoshitaka Utsumi, Toshitaka Nagao, Takao Itoi

**Affiliations:** 1 13112 Department of Gastroenterology and Hepatology, Tokyo Medical University, Shinjuku-ku, Japan; 2 Department of Gastroenterological Endoscopy, Tokyo Medical University, Shinjuku-ku, Japan; 3 13112 Department of Anatomic Pathology, Tokyo Medical University, Shinjuku-ku, Japan

Situs inversus totalis (SIT) is relatively rare, with an incidence of 1 in 10.000 individuals 1 . SIT is characterized by mirror-image transposition of the thoracoabdominal viscera. Endoscopic submucosal dissection (ESD) of early gastric cancer with SIT has been performed with the patient in the right lateral position to avoid submersion 2 3 ; however, a different standing position for the endoscopist as well as different positioning of the peripheral equipment are required. The use of gel during endoscopic treatment to overcome anatomical challenges and improve the visual field has been reported 4 5 . We report successful ESD of early gastric cancer with SIT during which scope maneuverability and the visual field were improved under low intraluminal pressure via water and gel immersion ( **Video 1** ).

Underwater endoscopic submucosal dissection using gel immersion for early gastric cancer with situs inversus totalis.Video 1

An 80-year-old man with SIT ( Fig. 1 ) presented with early gastric cancer (10 mm, type 0-IIa) on the posterior wall of the antrum on the gravitational side ( Fig. 2 ). Approaching the lesion was difficult because the lesion was submerged in gastric fluid and scope maneuverability was poor. Therefore, we removed gas from the lumen after marking the lesion, and filled it instead with water and gel ( Fig. 3 **a–d** ). The water and gel mixture provided lower intraluminal pressure with a clear view that allowed us to approach the lesion ( Fig. 3 **e, f** ). When the visual field was poor because of hemorrhage and mucus during mucosal incision, the addition of gel resulted in a clear view ( Fig. 3 **g–k** ). The underwater conditions provided buoyancy and aided the approach to the submucosal layer, resulting in successful en bloc resection ( Fig. 3 **l–o** ). The histopathological findings indicated curative resection ( Fig. 4 ).

**Fig. 1:**
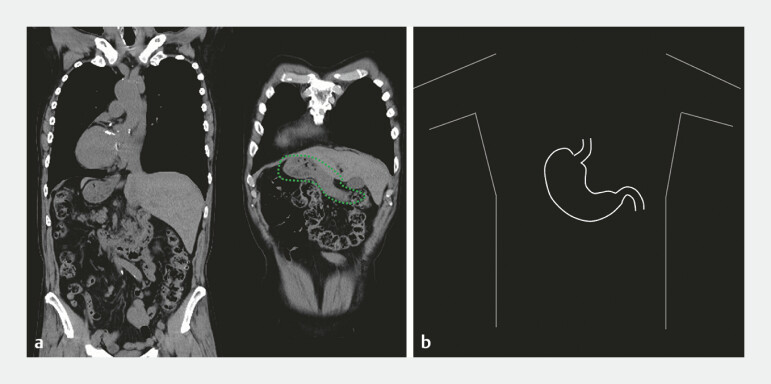
Situs inversus totalis (SIT). **a** Computed tomography image of complete mirror-image transposition of the thoracoabdominal viscera. The stomach is indicated by a green dotted circle. **b** Schema of the stomach with SIT.

**Fig. 2:**
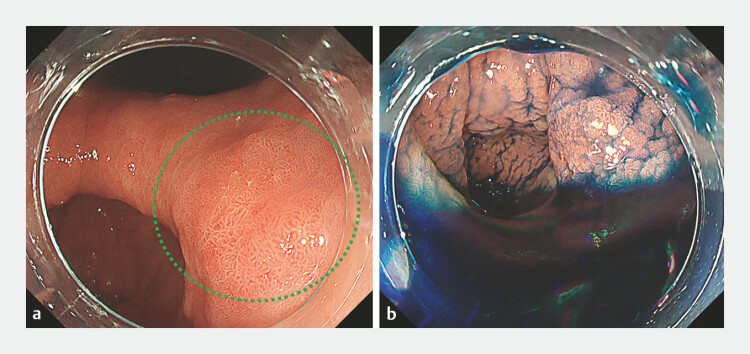
Endoscopic images. **a** White-light image. A flat elevated lesion (0-IIa) located on the posterior wall of the antrum (lesion diameter: 10 mm) was revealed by upper gastrointestinal endoscopy (green dotted circle). **b** Image with indigo carmine showing the antrum on the gravitational side and the submerged lesion.

**Fig. 3:**
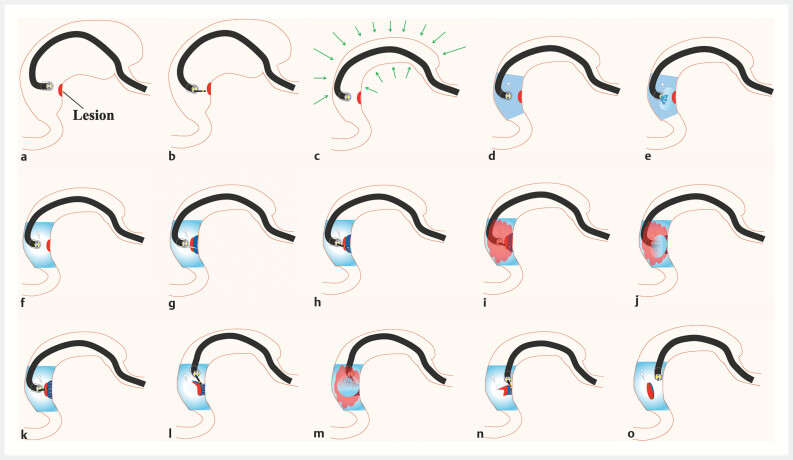
Underwater endoscopic submucosal dissection (ESD) with gel immersion for situs inversus totalis (SIT). **a** View of the lumen filled with gas. **b** Marking around the lesion. **c** Removal of gas from the lumen. **d** Underwater view. **e** Clear view after gel injection. **f** View of immersion under water and gel. **g** Local injection. **h** The initial mucosal incision from the distal edge of the lesion to the end point. **i** Bleeding during creation of the mucosal incision. **j** Gel injected due to poor endoscopic view. **k** Reduced bleeding and clear view obtained with gel immersion, allowing complete circumferential incision. **l** Submucosal dissection. **m** Gel added during the procedure as needed to maintain a clear field of view. **n** Underwater conditions, creating buoyancy and traction, support submucosal dissection. **o** Complete en bloc resection.

**Fig. 4:**
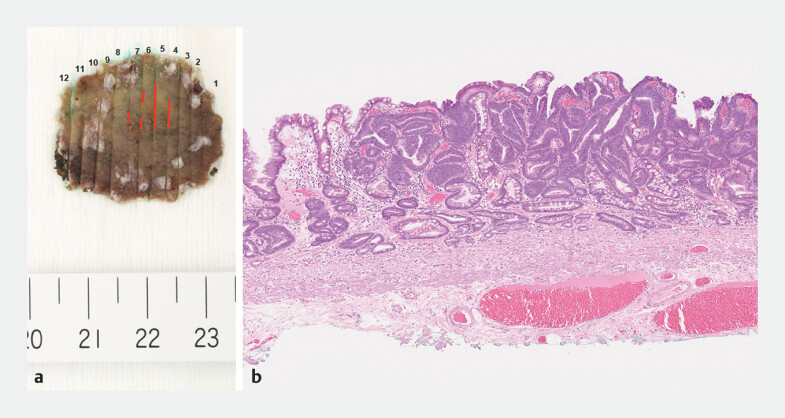
Macroscopic and histopathological images of the resected specimen. **a** Macroscopic image of the specimen. **b** Histopathological image of the specimen. The pathological diagnosis was intramucosal adenocarcinoma without lymphovascular invasion and with negative margins.

ESD of SIT using submersion via water and gel allowed safe resection under low pressure with improved maneuverability, as well as an improved visual field without changing the patient’s position.

Endoscopy_UCTN_Code_TTT_1AO_2AG_3AD

## Notes

Endoscopy E-Videos https://eref.thieme.de/e-videos
*E-Videos* is an open access online section of the journal *Endoscopy* , reporting on interesting cases and new techniques in gastroenterological endoscopy. All papers include a high-quality video and are published with a Creative Commons CC-BY license. Endoscopy E-Videos qualify for HINARI discounts and waivers and eligibility is automatically checked during the submission process. We grant 100% waivers to articles whose corresponding authors are based in Group A countries and 50% waivers to those who are based in Group B countries as classified by Research4Life (see: https://www.research4life.org/access/eligibility/ ). This section has its own submission website at https://mc.manuscriptcentral.com/e-videos .
